# The Unqualified Assistant: The Other Side

**Published:** 1897-12-11

**Authors:** 


### The Hospital

A Journal of

The Medical Sciences and Hospital Administration.

Vol. XXIII.—No. 585.] Edited by Sir Henry Burdett, K.C.B., and Solomon C. Smith, M.D., M.R.C.P. [Dec. 11, 1897.

### The Unqualified Assistant: The Other Side.

While we have every sympathy with the desire apparently felt by a majority of the General Medical Council to do away with the unqualified assistant, we cannot but admit the force of many of the protests which are beginning to pour in against their action in declaring that any practitioner who is proved to have employed an unqualified assistant "to attend or treat patients in respect of matters requiring professional discretion or skill" shall be "liable to be judged as guilty of 'infamous conduct in a professional respect,' and to have his name erased from the Medical Register." On the face of it the resolution is right; the difficulty comes in when we consider the actual conditions under which ordinary general practice has to be conducted. It is pointed out that where in the most perfect good faith a "surgery attendant" is employed to dispense and keep the books, it must happen that, if he is not allowed to run to an urgent call, that emergency will be attended to by some one else far less qualified than he would be.

The case is put in this way by one writer who has, as his "surgery attendant," a person who has gone through his full curriculum but has not become qualified. "Am I," he says, "to understand that if word is sent to my house that one of my patients is in the position of imminent danger from miscarriage, hæmorrhage, precipitate labour, or accident, during the absence of myself or assistant, such a competent 'attendant' is to shut the door and decline to act in the emergency, while the herbalists and midwives in the district, and the very policemen in the street, or some factory operative who may have attended a superficial course of ambulance lectures, are fit and proper persons to interfere in the hundred and one cases which arise in the everyday life of our manufacturing towns?" Such is the question. There can be no doubt that the case exactly falls within the definition given by the Medical Council, and that any medical man who allows his "surgery attendant" to go out to such an emergency will be at the mercy of any professional "brother" who chooses to report him. It is clear that, if this notice is put in operation impartially all round, it will have the effect of throwing an enormous amount of work into the hands of quacks, for the employment of qualified men as mere surgery attendants is out of the question; while if it is not put into operation all round its action will be most unfair. We are almost tempted to ask what will be the operation of the notice on those who employ nurses as their assistants, and whether the threatened action will not put some of our professional leaders in jeopardy every day, for it certainly is true that some of the work done by the nurses "employed as assistants" by operating surgeons of great eminence partakes much of the character of what is done in hospitals by house surgeons, and it may fairly be questioned whether some of the duties thrown on such nurses do not "require professional discretion or skill." This, of course, did not matter so long as anyone could employ unqualified assistants; but it is a matter of serious importance if Sir Somebody Something is to be allowed to "employ" his "assistant," who happens to be a nurse, to pass a catheter at night rather than that he should be disturbed at dinner, while poor Mr. Somebody Else, who has been working all day, may not, on a snowy night, send out his unqualified, but fully taught, assistant to do exactly the same thing.

Another point which is being urged by those who object to the action of the General Medical Council is, that while the unqualified medical assistants who are to be turned adrift at the bidding of the Council will merely step across the way and set up in competition with their late employers, the Council has refused, and continues to refuse, to take any steps to interfere with or prevent such unqualified practice. It is held that the abolition of the unqualified assistant should go hand in hand with the abolition of unqualified practice, and that if this is not done the action of the Council merely means so much into the pockets of the quacks. It is pointed out that in an epidemic of, say, diarrhœa, while the doctor, who is being run off his feet, dare not allow his trained, but unqualified, "surgery attendant" to give a bottle of physic to an urgent case, for fear of being turned off the Register by the Council, this Council stands idly by while these urgent cases are taken in hand and medically treated by all the chemists and quacks of the district, and thus become their "patients," not in emergencies only, but in all ordinary cases. The more the Council endeavours to put in force its powers the more obvious does it become that its powers must work much injustice, unless they are so extended as to interfere effectively with unqualified practice of all kinds.

